# High-Temperature Liquid Chromatography and the Hyphenation with Mass Spectrometry Using High-Pressure Electrospray Ionization

**DOI:** 10.5702/massspectrometry.S0079

**Published:** 2019-08-26

**Authors:** Lee Chuin Chen

**Affiliations:** 1 Interdisciplinary Graduate School of Medicine and Engineering, University of Yamanashi, 4–3–11 Takeda, Kofu, Yamanashi 400–8511, Japan

**Keywords:** high-temperature liquid chromatography, high-pressure electrospray ionization, capillary liquid chromatography, subcritical water LC-MS

## Abstract

Increasing the operating temperature of the liquid chromatography (LC) column has the same effect as reducing the diameter of the packing particles on minimizing the contribution of *C*-term in the van Deemter equation, flattening the curve of plate height *vs.* linear velocity in the high-speed region, thus allowing a fast LC analysis without the loss of plate count. While the use of smaller particles requires a higher pumping pressure, operating the column at higher temperature reduces the pressure due to lower liquid viscosity. At present, the adoption of high-temperature LC lags behind the ultra-high-pressure LC. Nevertheless, the availability of thermally stable columns has steadily improved and new innovations in this area have continued to emerge. This paper gives a brief review and updates on the recent advances in high-temperature liquid chromatography (HTLC). Recent efforts of hyphenating the capillary HTLC with mass spectrometry *via* a super-atmospheric pressure electrospray ionization is also reported.

## PRINCIPLE OF HIGH-TEMPERATURE LIQUID CHROMATOGRAPHY

The concept and the implementation of high-temperature liquid chromatography (HTLC) operated at elevated temperature greater than 100°C has been around since the 1980s. Early description of the theoretical principle of HTLC was given by Antia and Horváth.^1)^ Their paper highlighted the improvement of speed and efficiency that is achievable by operating the liquid chromatography at high temperatures and compared it favorably with supercritical fluid chromatography. Reviews on the principles and implementation of HTLC are given by Greibrokk and Andersen (2003),^2)^ Guillarme and Heinisch (2005),^3)^ Vanhoenacker and Sandra (2006),^4)^ McNeff *et al.* (2007),^5)^ Teutenberg (2009),^6)^ and Carr *et al.* (2010).^7)^ Reviews on metal oxide-based stationary phases are given by Nawrocki *et al.* (2004),^8)^ and Teutenberg *et al.* (2009).^9)^ Here we update some of the recent developments on this topic. Important equations that are useful for the understanding and the design of HTLC and the ion source are also listed.

### Principle of LC with the partition chromatography

We begin our discussion with the concept of the partition chromatography developed by Martin and Synge,^10)^ which is the origin of modern liquid chromatography. Imagine a lineup of a large number of separatory funnels, labeled as F_1_, F_2_, …F*_M_*, each filled the same volume amount of a particular organic solvent. Next, pour the same amount of aqueous solvent containing solute A into funnel F_1_. We assume the aqueous and organic solvent are totally immiscible and the following “reaction” takes place in the separatory funnel. 
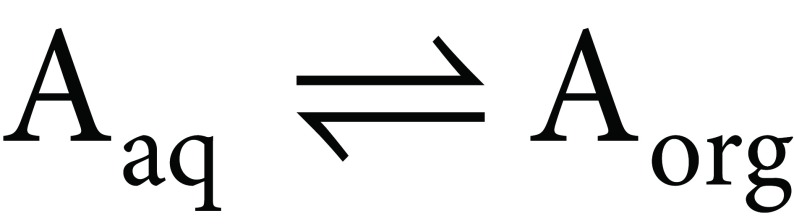
(1)

Based on the solubilities of A in different solvents, there is a net transfer of A from the aqueous (aq) phase to the organic phase (org) until the equilibrium is reached. The rate constant, *k_R_* for the forward and the reversed reaction rates is a function of temperature in the form of Arrhenius equation, *i.e.*, 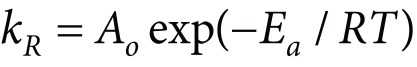
(2) where *E_a_* is the activation energy, *R* is the ideal gas constant, *T* is the absolute temperature, and *A_o_* is a constant, which means the reaction can be accelerated by raising the temperature. At equilibrium, the equilibrium constant *K* for this reaction is given by 
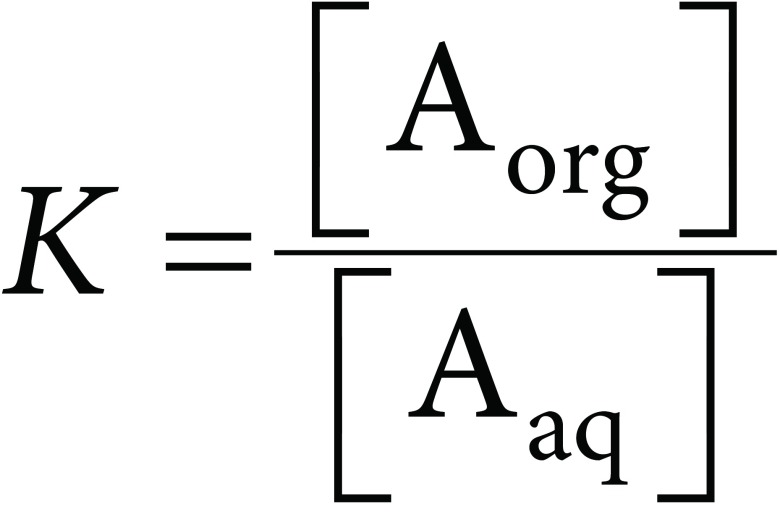
(3)

We label this first extraction step as *n*=1, and the fraction of the original amount of A in the mobile aqueous phase becomes 1/(1+*K*), and the fraction in the stationary organic phase is *K*/(1+*K*). In the next extraction step, *i.e.*, *n*=2, the aqueous phase in F_1_ is transferred to F_2_, and F_1_ is replenished with the same amount of fresh aqueous phase (without solute). Likewise, in the subsequent step, the aqueous phase in F*_m_* (if present) is transferred to F*_m_*_+1_ and F_1_ is replenished with new mobile phase. Let *f* be the fraction of A in the aqueous phase in funnel F*_m_* during *n*th extraction and it is given by: 
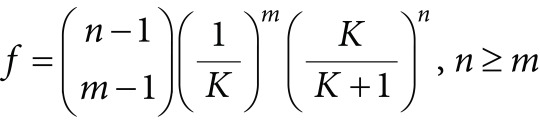
(4) where 
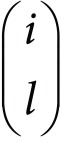
 is binomial coefficient, *m* is the funnel number, *n* is the number of extractions. This long lineup of separation funnels can be thought of as a chromatographic column and each funnel is a physical “theoretical plate” in which perfect equilibrium between the two phases takes place. To emulate the detection for a column with finite plate number *M*, assume a detector is placed at plate *m*=*M* to measure the solute fraction in the aqueous mobile phase for every *n*. The fraction will reach a maximum point at *n*=*n*_peak_, where (∂*f*/∂*n*)*_M_*_,_*_K_*=0. Solve (∂ ln *f*/∂*n*)*_M_*_,_*_K_*=0 by Stirling approximation gives 
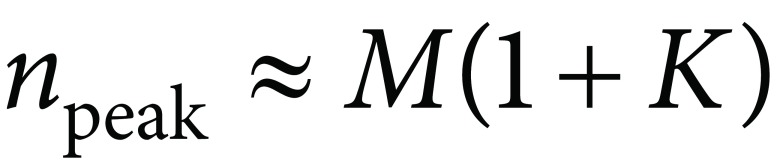
(5)

Thus, solutes with slightly different *K* can be separated with their fraction peak appears at different *n*. The separation and peak width are determined by the number of theoretical plates, *M*. If we ignore the time required to transfer the mobile phase, the overall speed depends on the time required to reach the equilibrium at each plate, which can be shortened by agitation, using a smaller amount of liquid volume, and by increasing the temperature of the system. Take note that the chemical equilibrium constant *K* follows Boltzmann distribution and is temperature dependent, *i.e.*: 
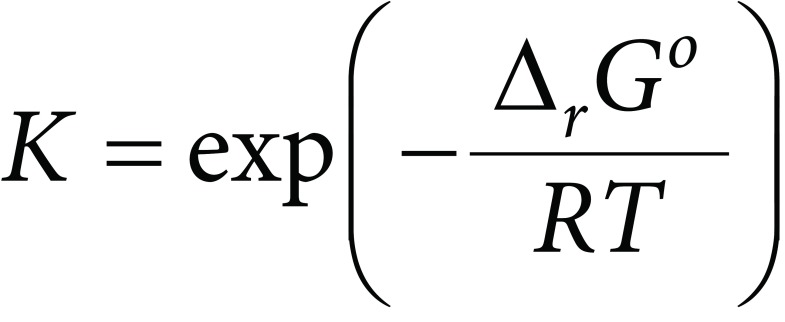
(6) where ∆*_r_G^o^* is the standard Gibbs energy of the reaction to change A from aqueous to organic phase.

In reality, it is impractical to perform LC with this imaginary apparatus because the operation is tedious and most importantly, it cannot achieve sufficient plate count with reasonable size. Instead of physical discrete plates, the first analytical liquid chromatography by Martin and Synge employed silica gel as the mechanical support for the stationary aqueous phase and used chloroform as the mobile phase.^10)^ To describe the column behavior, they applied the concept of “theoretical plates” in distillation which states that even though the equilibrium between the two phases is not established at any point, the column can be thought to be divided into a number of layers, each is equivalent to one “theoretical plate.” The plate height is defined as the thickness of that layer such that “solute concentration in the mobile phase leaving the layer is in equilibrium with the mean solute concentration in the stationary phase within the boundary of that layer.”^10)^ The success of partition chromatography gives birth to the paper and thin layer chromatography, and the modern gas and liquid chromatography. Historically, the “normal” configuration employed organic (hydrophobic) mobile phase, and aqueous (polar) stationary phase. Nowadays, the opposite configuration, the reversed phase chromatography with a hydrophobic stationary phase and a hydrophilic mobile phase is more widely used for general analytical applications.

### van Deemter equation

For a given length of the column, the smaller the plate height, the higher the number of “theoretical plate” for the use of separation. The plate height depends on diffusion and the flow rate of the mobile phase. Taking into account of the eddy diffusion, longitudinal diffusion, mass transfer of solute in the mobile phase and in the stationary phase, the performance of columns is described in term of plate height and linear velocity by van Deemter,^11)^ and in term of reduced plate height and reduced velocity by Knox.^12)^

Here we use the van Deemter equation for our discussion on the temperature effects, and the plate height, *H* is given by 
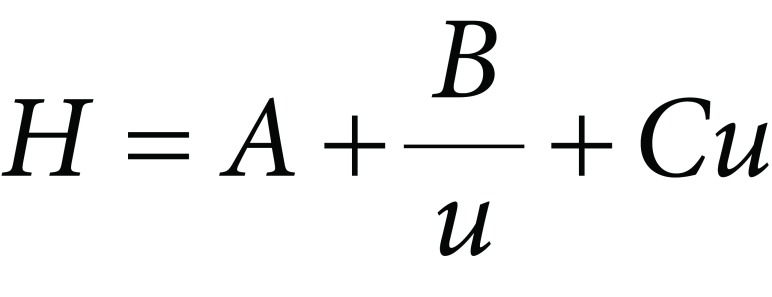
(7) where *u* is the linear velocity. The *A*-term is a parameter owing to the eddy diffusion caused by the multi-channeling through the packings. *B*-term is related to the axial diffusion and is proportional to the diffusivity coefficient of solute in the mobile phase, *D_m_*: 
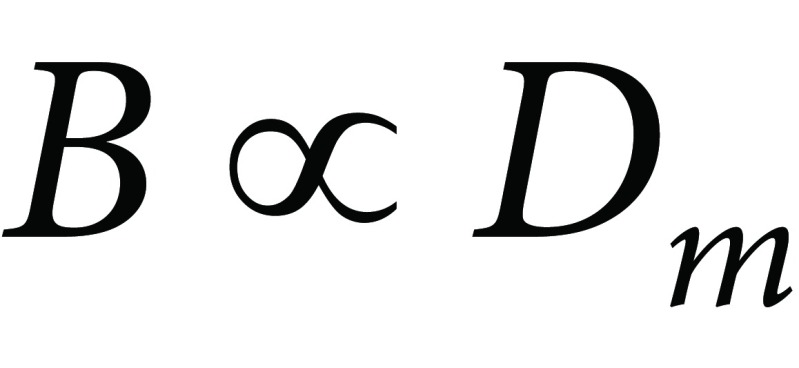
(8)
*C*-term is related to mass transfer and the finite time required to establish solute distribution in both phases and is proportional to the square of particle size and inversely proportional to *D_m_*, *i.e.*: 
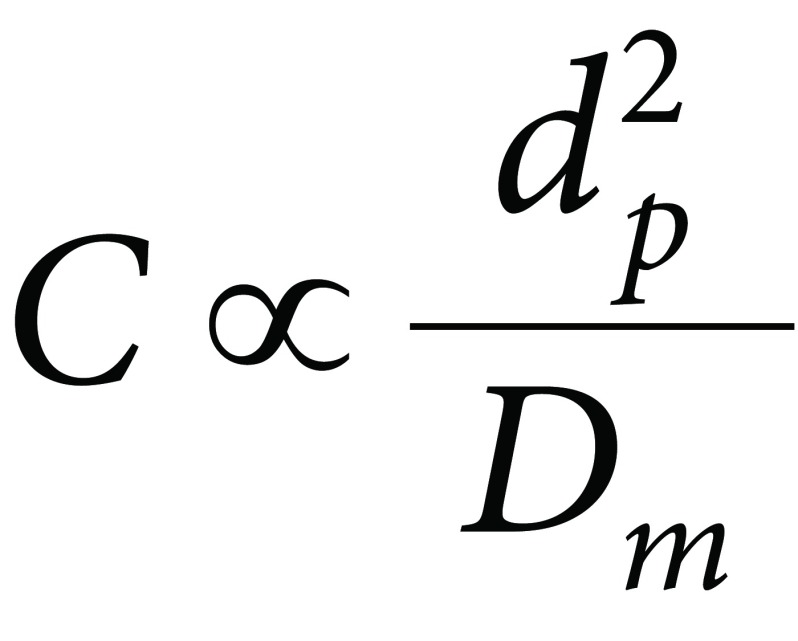
(9) where *d_p_* is the average diameter of the particles. The plot of plate height *vs.* linear velocity using non-zero *A*, *B*, and *C* shows a pattern like “し” in the Hiragana. Solve d*H*/d*u*=0 results in an optimum velocity: 
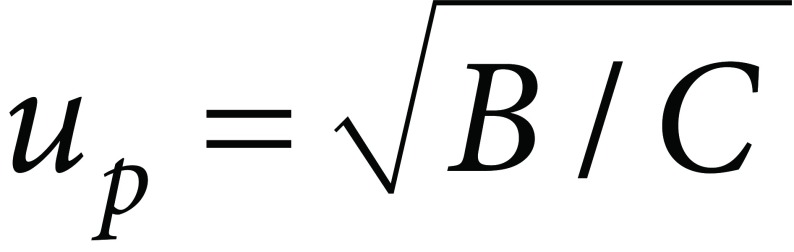
(10) at which the plate height, *H* is minimal. As indicated by Eqs. (8) and (9), the diffusion coefficient is an important parameter in modeling the performance of LC.

For the diffusion of spheres through a viscous fluid, the Stokes–Einstein equation gives 
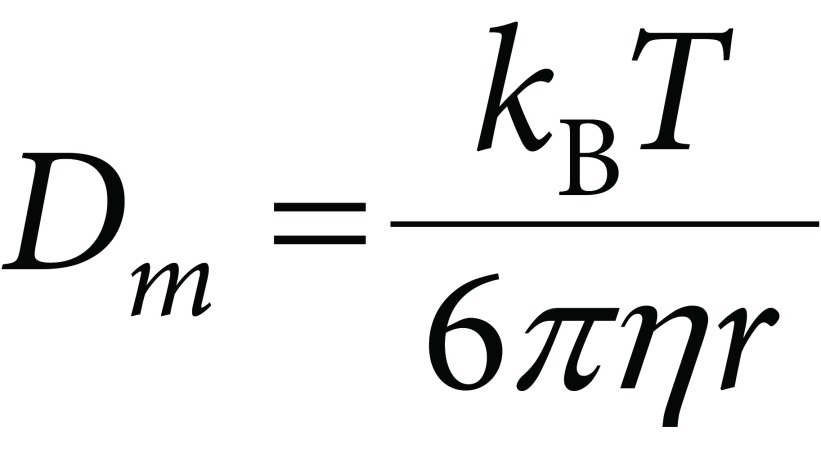
(11) where *k*_B_ is Boltzmann constant, *T* is the absolute temperature, η is dynamic viscosity, and *r* is the radius of the sphere. To fit the experimental results of organic solvents and solutes, a number of correlations are also available for estimating diffusion coefficient for dilute solutions.^13,14)^ A widely used empirical equation is the one by Wilke–Chang^14)^: 
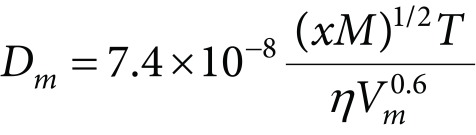
(12) where *M* is the molecular weight in g/mol, η is dynamic viscosity in cP, *T* is the absolute temperature in K, *V_m_* is molar volume in mL/mol, and *x* is solvent association factor. For water, *x*=2.6, and for nonassociated solvent, *x*=1. Substitute *B*∝(*T*/η) and *C*∝(ηd^2^*_p_*/*T*) into Eq. (10) yields 
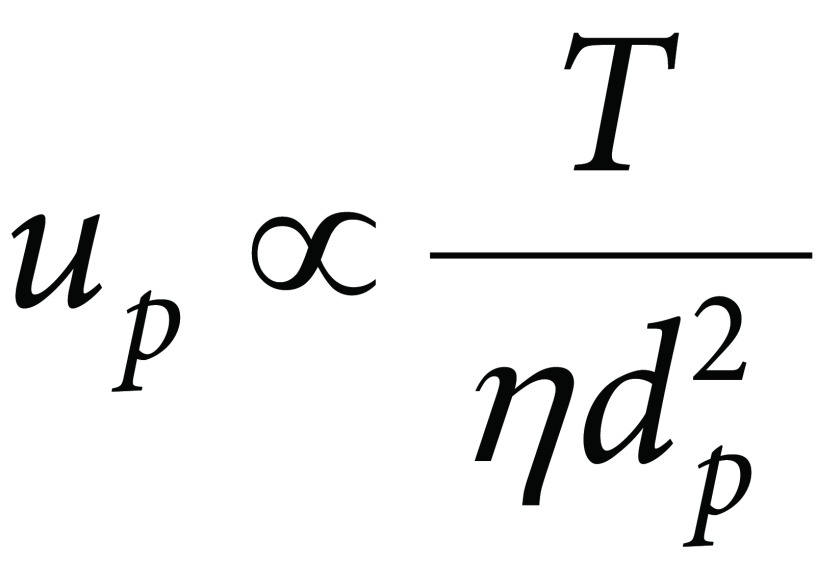
(13)

For a given particle diameter, the increase of temperature shifts the optimum velocity to a larger value. Note that viscosity is also a function of temperature and for liquid, it generally gets smaller with the increase of temperature.

At *u* greater than *u_p_*, the gradient d*H*/d*u*≈*C* and is proportional to *ηd_p_*^2^/*T.* That is, by increasing the temperature, d*H*/d*u* becomes smaller. In other words, van Deemter curve in *u*>*u_p_* region becomes flattened, which means the HTLC can be operated at high mobile phase velocity without sacrificing the plate height. It is noted that decreasing the diameter of the particles *d_p_* has a similar effect as that of increasing the temperature and that is the basis for improved performance of the ultrahigh pressure LC with sub-2 μm particle column.^15)^ Giddings pointed out that the analytical speed of GC is generally higher than a room temperature LC due to the large diffusivity of gases.^16)^ Carr *et al.* remarked that the improvement in diffusivity of liquid at elevated temperature can make the analytical speed of liquid chromatography on par with GC.^7)^

### Open tubular column chromatography

The *A*-term in the van Deemter equations is the result of the eddy diffusion, *i.e.*, multipath introduced by the packings when the molecule travels through the column. For an open tubular column which has only one pathway, the eddy diffusion, hence the *A*-term diminishes, leading to Golay equation: 
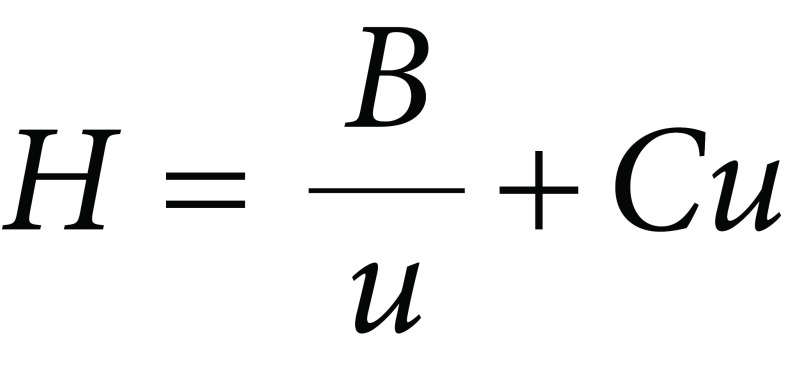
(14)

For this reason, modern GC uses open tubular rather than packed columns. The characteristic time for the solute at the center of the column to diffuse to the stationary phase on the wall is proportional to *r^2^/D_m_*, where *r* is the radius of the column. Diffusion in the liquid is much smaller compared to gas, thus, for open tubular columns to be used in LC, the inner diameter needs to be much smaller to achieve the equivalent effect in GC column. Recent reports used a narrow open tubular capillary with a diameter of 2 μm.^17,18)^ There were also efforts to increase the diffusion coefficient by operating the open tubular liquid chromatography (OT-LC) at elevated temperature to achieve the equivalent efficiency of narrow capillaries.^19)^ Coupling of high-temperature OT-LC to MS *via* APCI was also reported.^20)^

### van’t Hoff plot

The retention factor, *k* is defined as (*V_r_*−*V_o_*)/*V_o_*, where *V_r_* and *V_o_* are retention volumes of the retained solute and of an unretained “inert” reference. Under a constant flow rate operation, the retention factor is equivalent to the ratio of adjusted retention time of a retained peak to the time it takes for an unretained peak to travel through the column. The volume (*V_r_*−*V_o_*) equals the total volume of eluent needed to dissolve the solute, *V_E_*, *i.e.*, *k*=*V_E_*/*V_o_*. Under an isocratic condition, in which solvent composition remains constant throughout the elution, the equilibrium coefficient *K* equals *V_E_/V_s_*, where *V_s_* is the volume of the stationary phase. Since *V_o_* is the volume of mobile phase within the column, the retention factor *k* is connected to the thermodynamic equilibrium constant *K* by 
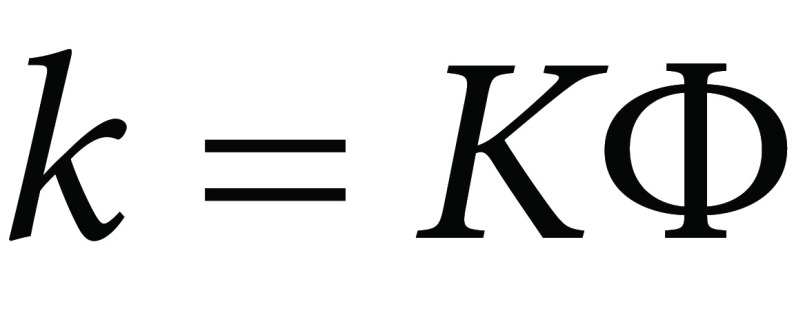
(15) where Φ is column phase ratio which is the ratio between the volumes of the stationary phase and the mobile phase. Φ is independent of analytes and is usually treated as constant during the analysis of LC performance. From Eqs. (6) and (15), we have: 
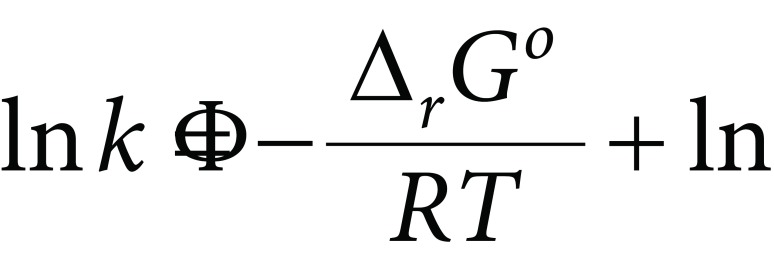
(16) or using thermodynamic relationship *G*=*H*−*TS*, the logarithm of retention factor is given by 
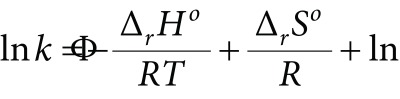
(17) where Δ*_r_H^o^* and Δ*_r_S^o^* are the standard enthalpy and entropy of the reaction for the retention, *i.e.*, for the transfer of a solute from mobile phase to the stationary phase.

For a given solute, the retention factor, or the retention time decreases with temperature. Theoretically, a plot of ln *k* *vs.* 1/*T* yields a straight line and the gradient gives the standard enthalpy. Examples of linear curves for HTLC can be found in Horváth (30–150°C),^21)^ Shen (150–200°C),^22)^ and Scott Kephart (90–370°C).^23)^ Nevertheless, non-linear van’t Hoff curve is also not unusual. Examples can be found in polymer column (150°C),^24)^ and hydrophobic interaction chromatography.^25)^ The mechanism of non-linear response is still subjected to continued investigation. Horváth introduced a temperature dependent enthalpy by adding a 1/*T*^2^ term to the van’t Hoff equation. There are also reports that evaluated the influence of the change in phase ratio (if there is any, due to the change of solvent composition, and temperature) to the non-linearity of van’t Hoff plot.^26,27)^

For the reversed-phase LC, there are three basic models for the retention mechanism: i) partitioning model in which solutes fully embed themselves into the bonded phase, ii) solvophobic,^28)^ and iii) adsorption model in which solutes are adsorbed only at the bonded phase/solvent interface. The actual retention process is circumstantial and it depends on the solutes, condition of the bonded phase (density, *etc.*) and the organic content of the mobile phase.^29–31)^ Recently, molecular simulations showed that the details of separation is indeed complicated and involved both partitioning and adsorption process.^32)^

### Solvent viscosity and the upper limit of linear velocity

**Figure 1:**
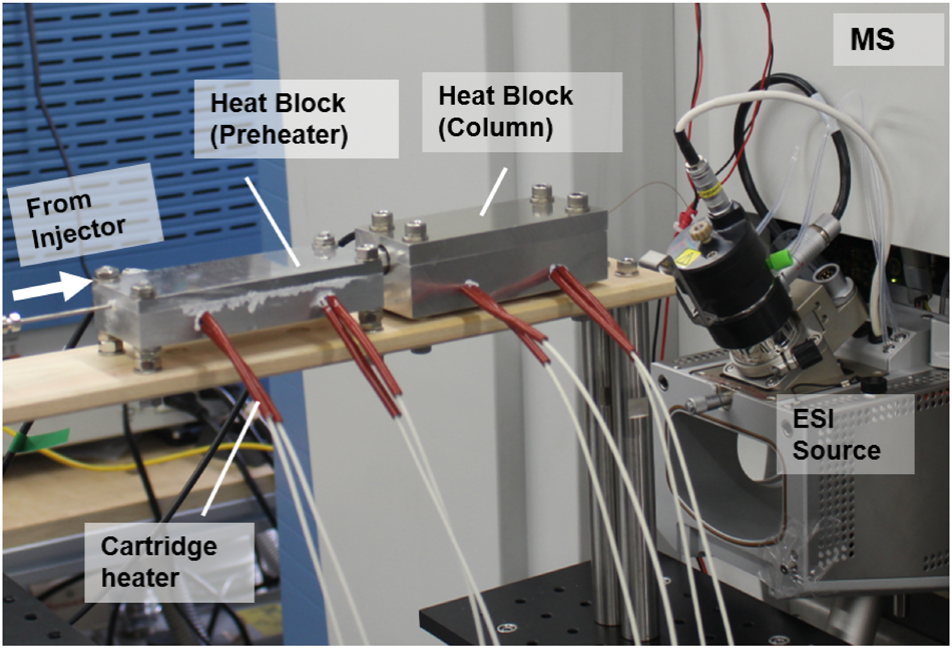
Fig. 1. Implementation of HTLC using a conventional atmospheric pressure ESI source for >100 μL/min operation. The heated column is connected to the ESI source *via* a fused silica capillary with 50 μm inner diameter that serves as a flow restrictor.

Besides being involved in the mechanism of diffusion and retention properties, viscosity is a key factor that set the upper limit for the available linear velocity of the mobile phase and hence the maximum speed of the analysis. Under a laminar flow regime, the volumetric flow rate, *Q* for an incompressible fluid across a circular tube is given by Hagen–Poiseuille equation: 
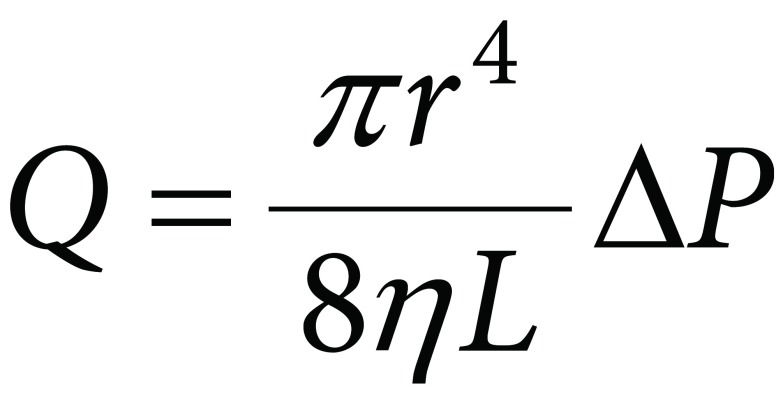
(18) where *r* is the tube radius, *L* is the tube length, η is the dynamic viscosity of the fluid, and Δ*P* is the pressure difference across the tube. Equation (18) is useful to estimate the pressure across the tube line in the LC system and the required radius for the design of flow restrictor. Figure 1 shows the implementation of HTLC using a 50 μm i.d. fused silica tube as a restrictor for a commercial ESI ion source. In most cases, the largest pressure drop takes place across the analytical column. The flow rate through the porous medium like LC column and porous rock is given by Darcy law: 
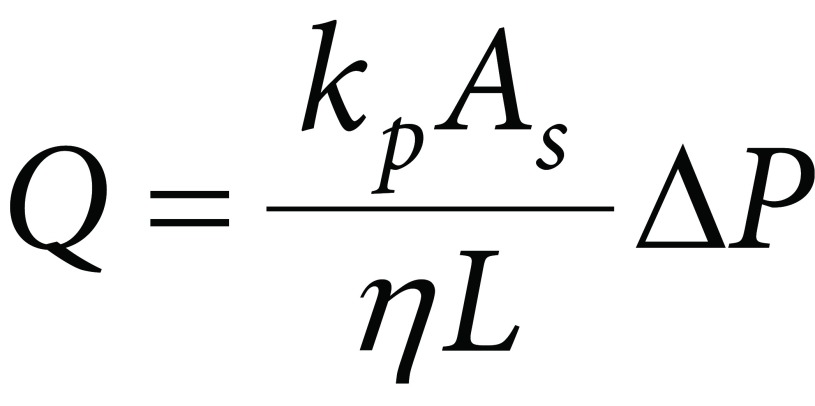
(19) where *k_p_* here is the permeability, and *A_s_* is the sectional cross area of the flow channel. For the column packed with spherical particles, the flow rate can be estimated using the Kozeny–Carman equation: 
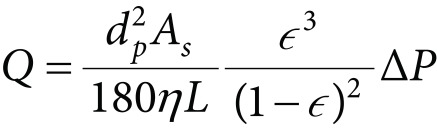
(20) where ϵ is the porosity *i.e.*, the volume of voids divided by the total volume, *d_p_* is the diameter of the particle. The porosity of packed columns is typically ∼0.4.^33)^

For a given column, viscosity determines the maximum volumetric flow rate that can be delivered by a particular liquid pump. There exist a number of equations for the viscosity–temperature relationship. A widely used equation is Vogel equation: 
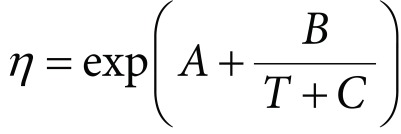
(21) where *A*, *B*, *C* are constants and *T* is the temperature in K. For viscosity in mPa·s or cP, Dortmund Data Bank gives *A*=−3.72, *B*=578.92, *C*=−137.55 for water. For acetonitrile, *A*=−3.16, *B*=459.98, *C*=−78.36.^34)^ For a binary solvent mixture, Grunberg–Nissan method can be used and the viscosity is given by^35)^

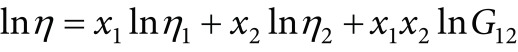
(22) where *x*_1_ and *x*_2_ are the mole fractions, and η_1_, η_2_ are viscosities for solvents 1 and 2. *G*_12_ is an interaction parameter which depends on the temperature and solvent components. For water/acetonitrile mixture, *G*_12_=exp(−1.762+929/*T*), for η in mPa·s.^36)^ Generally, the viscosity of liquid decreases with the increase of temperature. For example, the viscosity of water at 100°C is 0.28 times its value at 20°C.^37)^ For 50% v/v acetonitrile–water mixture, even a mild temperature at 60°C reduces the viscosity to half of its value at 20°C.^38)^ For organic/water solvent, the viscosity also increases with pressure.^39)^ HPLC liquid pump for column with particle diameter greater 2 μm usually has an upper limit for pressure at around 50 MPa. For particle diameter smaller or equals 2 μm, it needs the liquid pump that can withstand a backpressure up to 100 MPa. For a given pumping pressure, the reduction in eluent viscosity at elevated temperature allows one to operate the LC at a higher flow rate, or to use either longer columns or columns packed with smaller particles.

## IMPLEMENTATION OF HTLC

### Column materials

HTLC cannot be implemented without suitable column materials. Modern LC columns use silica as the packing materials and the stationary phases are chemically bonded on the silica surface. These columns are not intended for the operation at elevated temperature. The operating guides usually recommend an upper limit at 80°C. Pure silica has inherent instability at pH >7–8 and higher dissolution rates in aqueous medium at elevated temperatures. Stabilized silicas such as hybrid inorganic–organic particles have been developed to address these problems. Teutenberg *et al.* conducted a thermal durability test at 150°C on a number commercial silica-based reverse phase columns and shortlisted a few candidates for high-temperature operation.^9)^ XBridge C-18 was found to have the highest thermal resistant followed by Gemini NX C18. These columns utilize ethylene bridge to stabilize the silica-matrix which are effective to enhance their stability at high temperature.

One alternative to silica for high-temperature application is zirconia.^40,41)^ Zirconia bonded with polybutadiene (PBD) was reported to be equivalent to Octadecyl*-*silica (ODS, silica-C18 phase). Compared to silica, these particles are also stable under harsh pH conditions and have become commercially available recently. An extreme operating condition up to 300°C had been reported by Scott Kephart.^23)^ Using zirconia-based particles, the potential of HTLC for sub-minute analysis had been demonstrated by Carr group by flowing the eluent at 15 mL/min through a 50 mm long column with 4.6 mm inner diameter kept at 150°C.^36)^

Another commercially available heat resistive column is based on porous graphitic carbon (PGC). The column packed with 100% porous graphitic carbon is free of column bleeding (the elution of stationary phase) because these particles have no bonded phases. However, unlike the retention mechanism of the standard hydrophobic phases, the retention is due to the dispersive and charge induced interaction with the graphite surface.^42)^ Thus, the optimization of solvent composition and the selectivity can be different from that of C-18 column.^43)^ Besides hydrophobic compounds, PGC also retains polar compounds. A recent review on PGC stationary phase is given by West & Lafosse.^44)^ It is recently reported that modulation of the mobile-stationary phase of the carbon column increased its reproducibility and prevented the loss of retention.^45)^

Causon *et al.* used a polymeric monolithic column with pure water as eluent for the separation of *n*-alcohol up to 200°C.^46)^ The polymer material was poly(divinylbenzene). Commercial polymer column poly(styrene-*co*-divinylbenzene) was also used for the high-speed separation of proteins at a temperature as high as 140°C without protein degradation.^47)^ The finding was attributed to the short residence time proteins stayed within the heated column. The descriptions of these columns are summarized in Table 1.

**Table 1:** Table 1. Selected commercial columns.

Column	Manufacturer	Material	Tested temperature
XBridge C-18^a^	Waters	Organo-silica hybrid	150°C^9)^
Gemini NX C18^a^	Phenomenex	Organo-silica hybrid	150°C^9)^
ZirChrom-PBD^b^	ZirChrom	Zirconia	300°C^23)^
Hypercarb^b^	Thermo Fisher Scientific	Porous Graphitic Carbon	200°C^42)^
ProSwift^c^	Thermo Fisher Scientific (formerly Dionex)	Polymeric monolithic	150°C^47)^

^a^ Long-term durability not tested. ^b^ Well known thermal resistive column. ^c^ Operating temperature limited by the PEEK housing. PEEK has a glass transition temperature at 143°C.

### Column heater and heat-exchanger

Even not at the elevated temperature (>80°C), maintaining a constant temperature is needed to ensure the reproducibility of the analysis because the retention factor is a function of temperature. It is common to keep the column at a temperature above room temperature, *e.g.*, 40–60°C using an electric oven. Cryogenic operation was also reported to promote the retention of proteins.^48)^ For a typical column size with an inner diameter >2 mm, the mobile phase needs to be pre-heated before entering the column in order to achieve maximum efficiency.^49)^ Mismatching of the temperatures between the liquid and the column inner surface causes radial and axial temperature gradient which results in the broadening of chromatographic peaks.^50)^ In GC, the eluent gas is heated by forced convection of hot air bath. Liquids have much greater specific heat capacity than gas, thus heating the LC column using air bath is not efficient particularly at high eluent flow rate. An oil bath was used by Yan *et al.*^36)^ In their system, two separate heat exchangers were used to optimize the heating efficiency. One was a long heated tube (2.5 m) with a wider bore (i.d.: 0.03 in, o.d.: 1/16 in) for the eluent flowed at >10 mL/min. Another was a shorter tube (10 cm) with a smaller inner diameter (0.004 in i.d., 1/16 in o.d.) for the sample at 1 mL/min.

**Figure 2:**
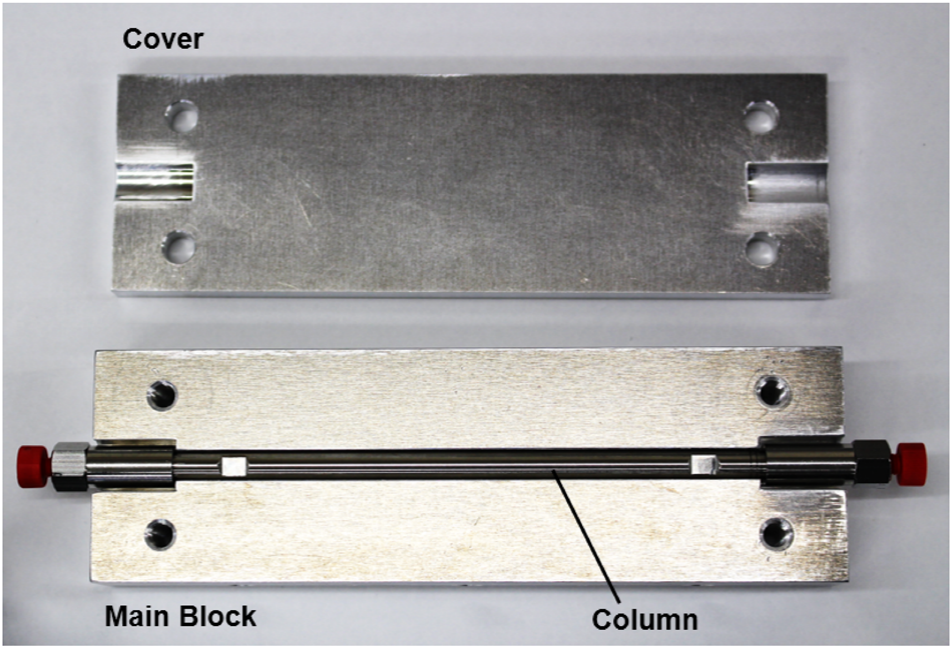
Fig. 2. Custom made heater block for the analytical column. The heat block is made of aluminum. The gap between the column and the heat block is filled with thermally conductive grease. The heat block is heated with two cartridge heaters.

In our system, the capillary column and the optional preheating tube were embedded within an aluminum block heated with two cartridge heaters and the gaps were filled with zinc-oxide based thermal grease (Fig. 2). For a circular heated tube with its flow axis at *z*-axis, we assume the liquid temperature is homogeneous radially and is a function of *z*, *i.e.*, it increases along the flow axis. In fluids, convection dominates the heat transfer. Assume the inner wall temperature of the heated tube is constant, the heat transfer rate for an infinitesimal tube length d*z* is thus: 
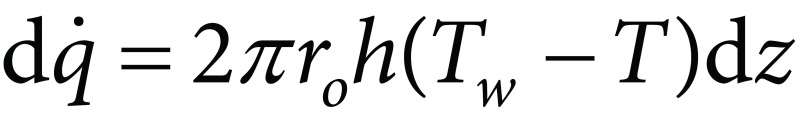
(23) where *r_o_* is the inner radius of the tube, *T_w_* is constant inner wall temperature, *h* is the heat transfer coefficient and *T* is the liquid temperature along the *z*-axis. For a fully developed laminar flow, convective heat transfer depends on the diameter of the tube and is given as *h*=*k_F_*Nu/*D*, where *k_F_* is thermal conductivity of the fluid, *D* is tube diameter and Nu is the Nusselt number. Nu=4.36 for constant surface heat flux. For water, *k_F_* is around 0.6 Wm^−1^K^−1^ at room temperature, thus *h* is approximately 26160 Wm^−2^K^−1^ for a narrow tube with 0.1 mm i.d. On the other hand, the heat transfer rate to raise the temperature of a flowing liquid is: 
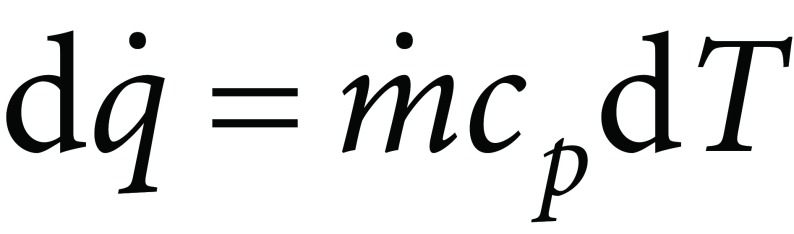
(24) where *ṁ* is mass flow rate, and *c_p_* is the specific heat coefficient of the fluid. Equate Eqs. (23) and (24) and solve the differential equation for *T*, we have: 
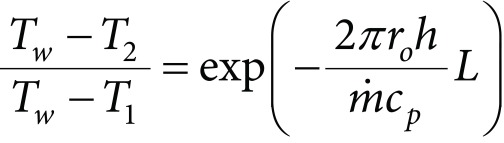
(25) or in term of flow velocity: 
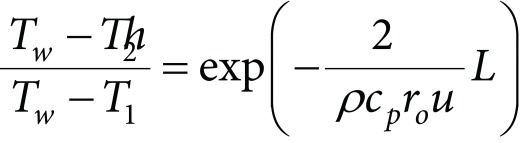
(26) where *T*_1_ and *T*_2_ are the liquid temperatures at the inlet and outlet of the heated tube, *L* is the length of the heated tube, ρ is the density of the fluid, and *u* is the linear velocity of the fluid. For a given fluid velocity, the design criteria are to keep *T_w_*−*T*_2_ under a certain value. Let’s say we are to operate the heat exchanger for water under the following conditions: *T*_1_=25°C, *T*_2_=195°C, *T_w_*=200°C, *r_o_*=0.05 mm, liquid flow rate=10 μL/min. The linear velocity is 21 mm/s, and the minimum required length, *L* for the heated tube is 0.3 mm. The small value of required length means that additional pre-heating loop for the eluent is unnecessary for low flow rate operation in the capillary LC, and the eluent is rapidly heated when it enters the heated column connector. The same also applies to chip-based HTLC with narrow columns.^51)^

### Capillary HTLC

Capillary LC employs narrow-bore column in the form of a packed column or monolithic column. The inner diameter is less than 0.5 mm, and the flow rate is in the order of μL/min compared to the mL/min for the column with i.d. >2 mm, The linear velocity at 10 μL/min through a 0.1 mm i.d. capillary LC is equivalent to flowing a mobile phase at 20 mL/min through a standard 4.6 mm i.d. column. As discussed in the heat exchanger section, the low mass flow rate in capillary LC columns allows rapid heating of the mobile phase, therefore a pre-heating process is not needed for their operation at high temperature. Column heater alone is enough for capillary HTLC.

Temperature programmed HT-capillary LC was demonstrated by Trones *et al.* using a capillary column which was packed with reversed phase material (Octadecyl-silica, ODS) using supercritical carbon dioxide as the slurry fluid.^52)^ As shown in Eq. (17), changing the temperature alters the analyte retention factor, thus produce a similar effect of changing the solvent gradient. For the chip-based HTLC, it was easier to improve the separation of the analytes using temperature gradient compared to the solvent gradient.^53)^ A high-speed separation using chip-based HTLC employing rapid heating up to 200°C and forced convection cooling was also reported.^51)^ Using polymeric monolithic capillary column, HTLC with pure water as a mobile phase has also been performed for the separation of *n*-alcohols at 200°C.^46)^

### Detectors

#### UV detector

The common detection method for HTLC is UV detection. UV detector with a back-pressure regulator was used to prevent the boiling and the rapid evaporation of the mobile phase when it exits the column.^36)^ The hot eluent from the column outlet also needs to be cooled to a temperature that is safe for the UV detector.

#### Flame ionization detection *etc.*

Flame ionization detectors are used widely in GC for the detection of organic substances. It is not coupled directly to LC because the flammable organic solvent would produce too many ions that saturate the detection system. Coupling of FID to LC thus far involved an intermediate or transfer stage that used moving belt, wire, or chain to removes the organic solvent first before feeding to the FID detector.^54)^ For HTLC that uses pure water (without organic content) as the mobile phase, FID had been coupled to the FID directly.^23,55)^ For compounds that do not absorb UV light, other detection methods such as evaporative light scattering detection (ELSD),^56)^ nuclear magnetic resonance (NMR),^57)^ Fourier transform infrared spectroscopy (FTIR),^58)^ and IR absorbance and scattering detectors are also available.^59)^

#### Mass spectrometry

The coupling of HTLC to MS had been performed using pneumatically-assisted ESI or APCI at >100 μL/min and with the use of flow restrictors of 50–64 μm.^42,60)^ One such configuration is shown in Fig. 1. The pressure drop across the flow restrictor (owing to the Hagen–Poiseuille law) under the flow regime of several hundreds of μL/min was enough to provide a back pressure to the column to avoid the boiling of eluent. Although the conventional flow regime LC-MS is in the order of several hundred μL/min, the efficiency of ESI and its tolerance to contaminants is higher under lower liquid flow rate.^61–63)^ Spraying samples sparingly at a few μL/min or less is ironically more sensitive than introducing a large amount of sample in a short time. Although flow splitters can be used to divert the extra eluent to waste, it would be economical and more sensitive to employ μL/min flow rate for the whole LC and ESI system to reduce the sample and solvent consumption and matrix effects.^64,65)^ Technically, low flow rate HTLC can be coupled to micro or nanoESI *via* a flow restrictor but the back pressure generated by restrictor depends on the viscosity and varies with the liquid flow rate. Under the flow regime of several tens or several μL/min and with the reduced viscosity at high temperature, the pressure drop across the restrictor may be insufficient for elevated temperature (>120°C) operation. The addition of a restrictor and cooling loop also introduces an additional dead volume to the system that causes the post-column broadening. To address this issue, direct coupling of capillary HTLC to ESI was performed in our laboratory using a high-pressure ion source.^66)^

## HYPHENATION OF HTLC WITH ESI-MS

### High pressure (>1 atm) electrospray

The onset potential to initiate electrospray, *V_ES_* is proportional to the square root of surface tension γ of the liquid: 
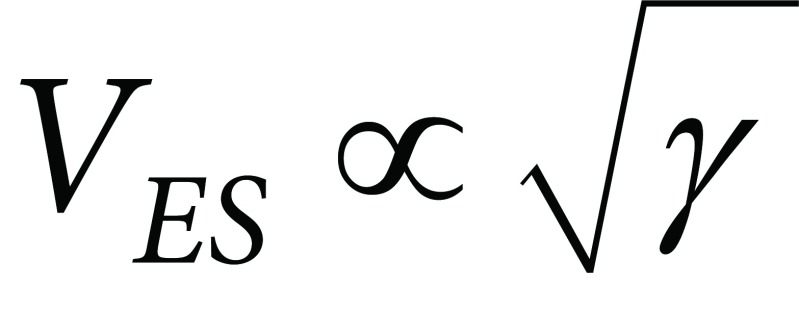
(27)

The choice of solvent thus has a great effect on *V_ES_* because the value of surface tension varies significantly from one liquid to another. For example, the surface tension for methanol is 22.7 mN/m at room temperature, whereas, for water, it is 72.8 mN/m; thus, *V_ES_* for water is nearly double that for methanol. Without pneumatic assistance, a pure aqueous solution is notoriously difficult to spray because it requires a higher potential to overcome the surface tension, and the gaseous break down that leads to the arcing or unwanted corona discharge can take place first before the onset of stable electrospray. The breakdown voltage between electrodes in a gas is a function of the product of the distance and the pressure. The threshold potential for breakdown, *V_B_* for two wide and flat electrodes is given by Paschen law as 
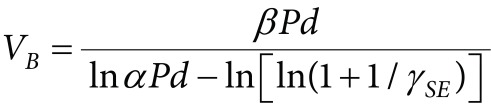
(28) where *P* is pressure, *d* is the distance between the electrodes, α and β are constants, and γ*_SE_* is the secondary electron emission coefficient. *V_ES_* for electrospray is not affected by gas pressure over the range of vacuum to 2 MPa (∼20 atm). On the other hand, *V_B_* increases significantly with the gas pressure surrounding the electrodes. Increasing the ESI operating pressure, therefore, can shift *V_B_* to a value larger than *V_ES_*, allowing a stable electrospray of large surface tension liquid such as pure water without discharge. Applying this principle, the high-pressure ESI (HPESI) source with an operating pressure greater than 1 atm has been developed in our laboratory to prevent the unwanted electrical discharge when dealing with pure water solution and to increase the desolvation efficiency.^67–70)^ Under a moderate pressure of <0.7 MPa, direct coupling to mass spectrometer can be performed with a modified ion transfer tube without the addition of extra pumping. Compared to a solution prepared in organic solvents, ion abundance of hydrophobic contaminants was reduced and much cleaner spectrum could be obtained using pure aqueous solution.^68)^

Raising the liquid temperature under atmospheric pressure is limited by its normal boiling temperature, *e.g.*, 100°C for water. Excluding the effect of superheating, the boiling of liquids takes place when the vapor pressure reaches the ambient pressure, so the boiling point for a given ambient pressure can also be estimated from the vapor pressure–temperature relationship. The correlation of vapor pressure and the temperature is described with good accuracy by the Antoine equation: 
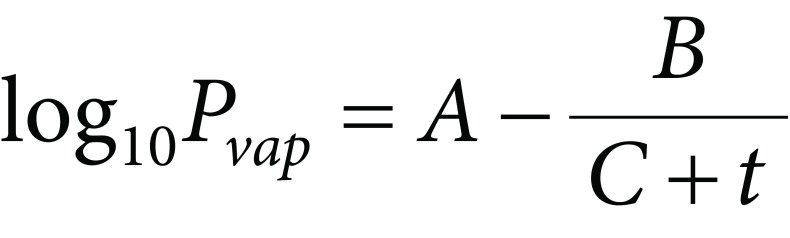
(29) where *P_vap_* is vapor pressure in mmHg, and *t* is temperature in °C. The constants *A*, *B*, and *C* for a variety of organic and inorganic solvents are given in literature.^71)^ For water, the Dortmund Data Bank gives *A*=8.14019, *B*=1810.94, *C*=244.485 for *t* ranging from 99 to 374°C.^34)^ The boiling point of a particular liquid can be raised by compressing the liquid container.

**Figure 3:**
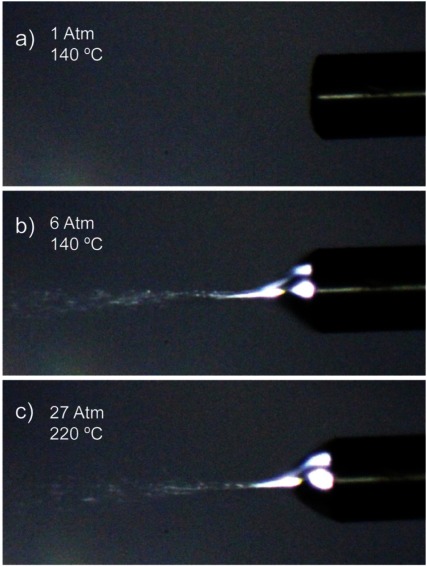
Fig. 3. Photograph of electrospray different pressures and emitter temperatures. (a) 1 atm and 140°C, (b) 6 atm and 140°C, and (c) 27 atm and 220°C. The solution is pure water and the flow rate is 4 μL/min. No electrospray can be seen in (a) because of the complete evaporation of water. Reproduced with permission from J. Mass Spectrom. (2016), 51, 396–411.^73)^

Using a super-atmospheric pressure ESI source, we have previously demonstrated a stable electrospray of solutions with the liquid temperature at 220°C under 2.2 MPa (∼22 atm).^72)^ Figure 3 shows the electrospray of water under different pressures and temperatures. Under atmospheric pressure, the electrospray of pure water was unstable owing to corona discharge. However, the formation of a water droplet at the ESI emitter could be observed up to ∼100°C under a liquid flow rate of 4 L/min with occasional bumping and bursting of the bulk droplet. When the emitter temperature was set to 140°C, the water droplet disappeared due to the complete evaporation (Fig. 3a). The electrospray emerged from the emitter tip (Fig. 3b) after pressurizing the ion source to 6 atm with compressed air. Under 2.7 MPa (∼27 atm), the boiling point of water was approximately 228°C and the observed electrospray was stable up to 220°C (Fig. 3c). For the HTLC column that was connected directly to a high-pressure ESI source, the column could be operated up 160°C under a moderate pressure of 0.7 MPa.^66)^

#### Construction of high-pressure ion source of HTLC

**Figure 4:**
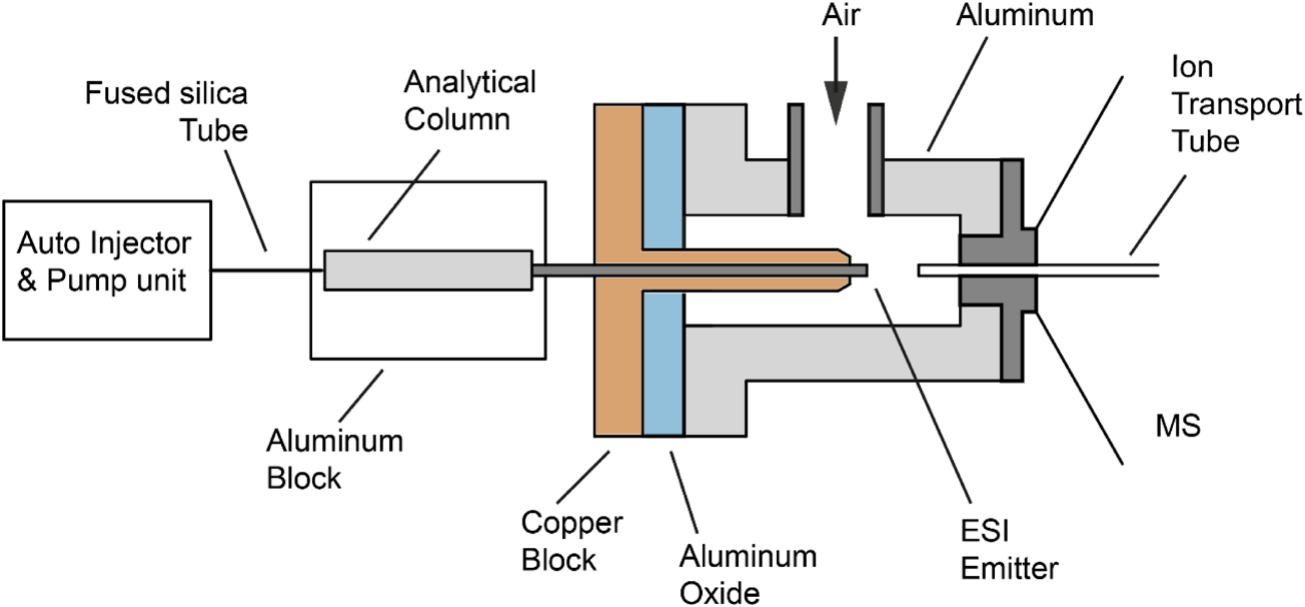
Fig. 4. Schematic of the hyphenation of high-temperature capillary LC with high-pressure ESI-MS. The ion source is pressurized with air from a compressor. The fused silica tube from the injector is connected directly to the analytical column.

**Figure 5:**
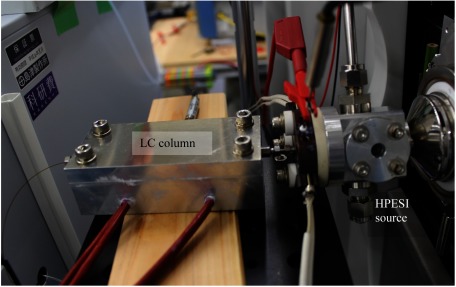
Fig. 5. Photograph showing the configuration of the HTLC-HPESI-MS using a capillary column (10 cm×0.1 mm i.d). The preheater is not in use. The column is embedded within the aluminum block.

The schematic of the high-pressure ESI source for high-temperature applications was shown in Fig. 4. The photograph of the constructed ion source is shown in Fig. 5. The ESI emitter (stainless steel capillary of 0.1 mm i.d. and 0.2 mm o.d. from Nilaco, Tokyo, Japan) was housed inside an ion source chamber made of aluminum alloy. To keep the ESI emitter at a constant temperature, the ESI capillary was embedded within a copper heater block with temperature control. The ion source was pressurized with air from an air compressor and the ion source pressure could be varied depending on the column temperature. The maximum pressure was 0.7 MPa (absolute pressure). HTLC was coupled directly to the HPESI without using flow restrictor and post-column cooling. Under a super-atmospheric pressure operation, the ion source itself functioned as the pressure regulator to the LC system to prevent the liquid from boiling within the column.

#### Coupling of ion source to mass spectrometer

We have adopted a number of strategies to couple high-pressure ion sources to commercial mass spectrometers depending on the objective, and the required gas throughput of the experiments. The details are discussed in our previous reports.^73–75)^ In the recent experiment with HTLC, a homemade ion transfer tube with 0.25 mm inner diameter was used to sample the generated ions inside the high-pressure vessel.^66)^

### Capillary HTLC-HPESI MS using subcritical water as mobile phase

**Figure 6:**
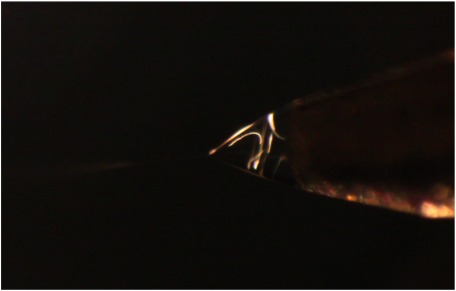
Fig. 6. Photograph showing the appearance of bubbles from the ESI capillary. The ESI is at near room temperature while the column is kept at 100°C. The cavitation stops when the ambient around the ESI emitter is pressurized to >1 atm.

Under the flow rate of several to several tens μL/min, the liquid was in good thermal equilibrium with the emitter capillary. Under 1 atm, the spray became unstable at 100°C emitter temperature and the bumping or bursting of liquid took place. Even by cooling down ion source, the boiling of eluent within the heated column still occurred if the backpressure was insufficient. In Fig. 6, the ion source was placed under atmospheric pressure and at room temperature while the column temperature was kept at 100°C. The bubbles owing to the cavitation of the eluent within the column can be seen emerging from ESI capillary into the liquid cone at the emitter tip. The bubbling eased when the ion source was pressurized with nitrogen or air.

To prevent the boiling, the conventional strategy to couple the HTLC to ESI-MS is to add a flow restrictor and a post-column cooler between them. While such an arrangement is suitable for >100 μL/min operation, the flow restriction is not appreciable for low flow rate and high-temperature operation and it introduces unnecessary dead volume to the system. For low flow rate operation, high-pressure ESI is a straightforward remedy to this problem. Pre-heating the eluent for the capillary column was unnecessary and removing the preheater capillary had actually reduced the dead time and pre-column dispersion.

For the liquid like water, the dielectric constant (polarity) also decreases with temperature,^76,77)^ and at elevated temperature, water behaves as if it is an organic solvent.^78)^ In LC, a change of column temperature has an equivalent effect of a change in acetonitrile concentration on retention.^21)^ This forms the basis for temperature programming as an alternative to gradient elution. A recent analysis showed that even for an isothermal operation, it was advantageous to set the temperature to the highest possible value that can be tolerated by the instrument and analytes to optimize the peak capacity.^79)^

Besides high throughput analysis, HTLC using superheated water has also emerged as a promising green LC method.^55,23,80)^ However, the elution strength of the subcritical water, though is much higher than that at room temperature, is still smaller than those of organic solvents. In practice, the elution using pure water for hydrophobic compounds takes a relatively long time to complete or needs to be operated at much higher flow rates using a conventional size column. The use of capillary column allows a high linear velocity while keeping the volumetric flow rate low.

**Figure 7:**
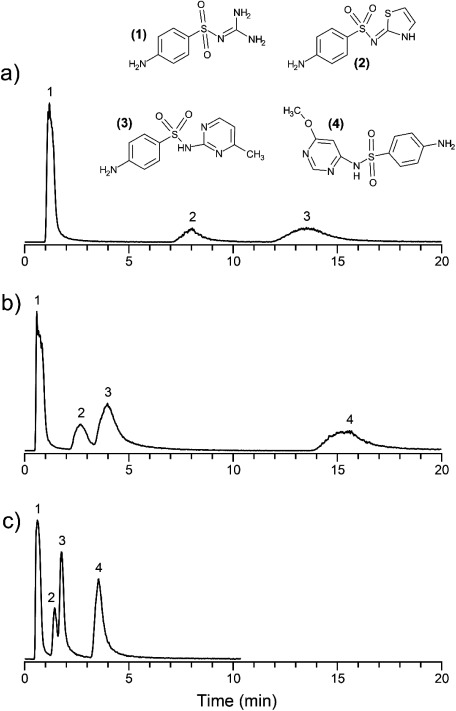
Fig. 7. Chromatograms obtained using pure water as the mobile phase at different column temperatures. Peaks are 1: sulfaguanidine, 2: sulfathiazole, 3: sulfamerazine, 4: sulfamonomethoxine. a) 80°C, b) 120°C, and c) temperature gradient from 120 to 150°C at 12°C/min. The flow rate of pure water is 10 μL/min. Reproduced with permission from Analyst (2018), 143, 5552–5558.^66)^

Figure 7 shows the subcritical water HTLC-HPESI MS of four sulfa drugs using a capillary column (10 cm long, i.d. 0.1 mm) with 5 μm porous graphitic carbon particles (Thermo Scientific) under isocratic conditions.^66)^ Pure water was used as the mobile phase and the flow rate was set to 10 μL/min. At this flow rate and with the column of 0.1 mm in inner diameter, the linear velocity (∼21 mm/s) of the mobile phase was equivalent to that with a flow rate of approximately 21 mL/min for a 4.6 mm column. The minimum temperature of the column was approximately 80°C in order to operate the LC system below 25 MPa. At 80°C, one of the compounds, sulfamonomethoxine was strongly retained and could not be eluted even with over one-hour elution time. Under an isothermal operation of 150°C, sulfathiazole (2), and sulfamerazine (3) were both eluted in less than 1.5 min, and could not be well resolved owing to the limited theoretical plate number of the column and the post column dispersion which had not yet been minimized. Temperature gradient strategy was employed to improve the separation of (2) and (3) by ramping the column temperature from 120 to 150°C at 12°C/min. The system pressure for 10 μL/min pure water flow rate at a column temperature of 150°C was 13 MPa, thus the further reduction in particle size or column i.d. is still possible.

**Figure 8:**
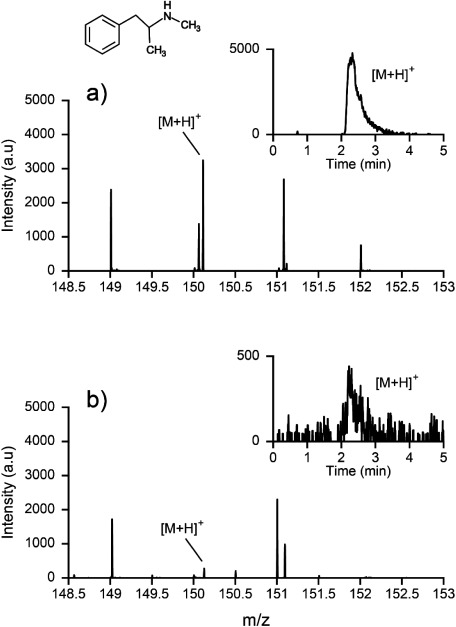
Fig. 8. Detection of 100 fmol methamphetamines in diluted raw urine by a) high-temperature capillary LC-MS (column i.d.: 0.1 mm) using HPESI under an operating pressure of 0.4 MPa, and b) high-temperature LC-MS (column i.d.: 1 mm) using pneumatically assisted ESI under atmospheric pressure. Column temperatures: 140°C, mobile phase: 9% (w/w) acetonitrile aqueous solution, solution flow rates: 10 μL/min for HPESI (a) and 400 μL/min for pneumatically assisted ESI (b). Insets show the EIC for the protonated methamphetamine. Reproduced with permission from Analyst (2018), 143, 5552–5558.^66)^

Operating the ESI at low volumetric flow rate can reduce the effect of ion suppression due to matrix effect and enhanced sensitivity owing to small precursor charged droplets. Compare to the large-bore column, capillary allows smaller amounts of sample to be detected with high sensitivity with ESI. One example is shown in Fig. 8 for the detection of 100 fmol methamphetamines prepared in diluted raw urine. The methamphetamine doped urine sample was injected directly without additional clean-up and the mobile phase used in this measurement was 9% (w/w) acetonitrile aqueous solution and the flow rate was 10 μL/min. The detection of the same amount sample using a standard pneumatically assisted ESI (as shown in Fig. 1) under atmospheric pressure is accompanied in Fig. 8b. To prevent the boiling of eluent, a flow restrictor of 50 μm i.d. was placed in between the column and ESI source and a flow rate of over hundred μL/min solution was necessary to create the sufficient back pressure to the column. Column preheater was also required in that operation. For a 1 mm i.d. column, the flow rate of 400 μL/min to obtain equivalent speed and retention time to that of capillary LC in Fig. 8a. The results clearly show the advantage of microflow capillary LC-MS in dealing with a small amount of sample.

## FUTURE PROSPECT AND CONCLUSION

### Thermally assisted non-enzymatic digestion

Thermally assisted non-enzymatic digestion of protein was observed in the 1950s when aspartic acid was preferentially released much sooner than other amino acids during the hydrolysis of proteins in diluted organic acids at boiling temperatures.^81,82)^ The phenomenon of selective cleavage of proteins at either or both sides of the aspartate residues (Asp, D), was believed to involve the ring closure between the carboxyl group of the aspartic acid and the adjacent carbonyl group in either direction.^81)^ The opening of the five-membered ring by H_2_O cleaves the polypeptide backbone on the C-terminal side, whereas the opening of the six-membered ring results in the cleavage on the N-terminal side of aspartate. Unlike enzymes, which cannot withstand high temperature, acidic hydrolysis can be accelerated by heating the liquid to >100°C and the non-enzymatic digestion acidic hydrolysis is gaining increasing attention for the bottom-up proteomics. Li *et al.* heated the diluted formic acid solution containing proteins to 108°C for 1–2 h and analyzed the digested products using mass spectrometry.^83)^ The digestion process had also been performed using a microwave oven to provide uniform heating to the polar solvent.^84–86)^ The digestion process that typically requires several hours to complete can be shortened to approximately 5–10 min by incubating the sample in a microwave oven. In those works, ESI-MS of the digestion products were performed separately after cooling the sample to near room temperature.

**Figure 9:**
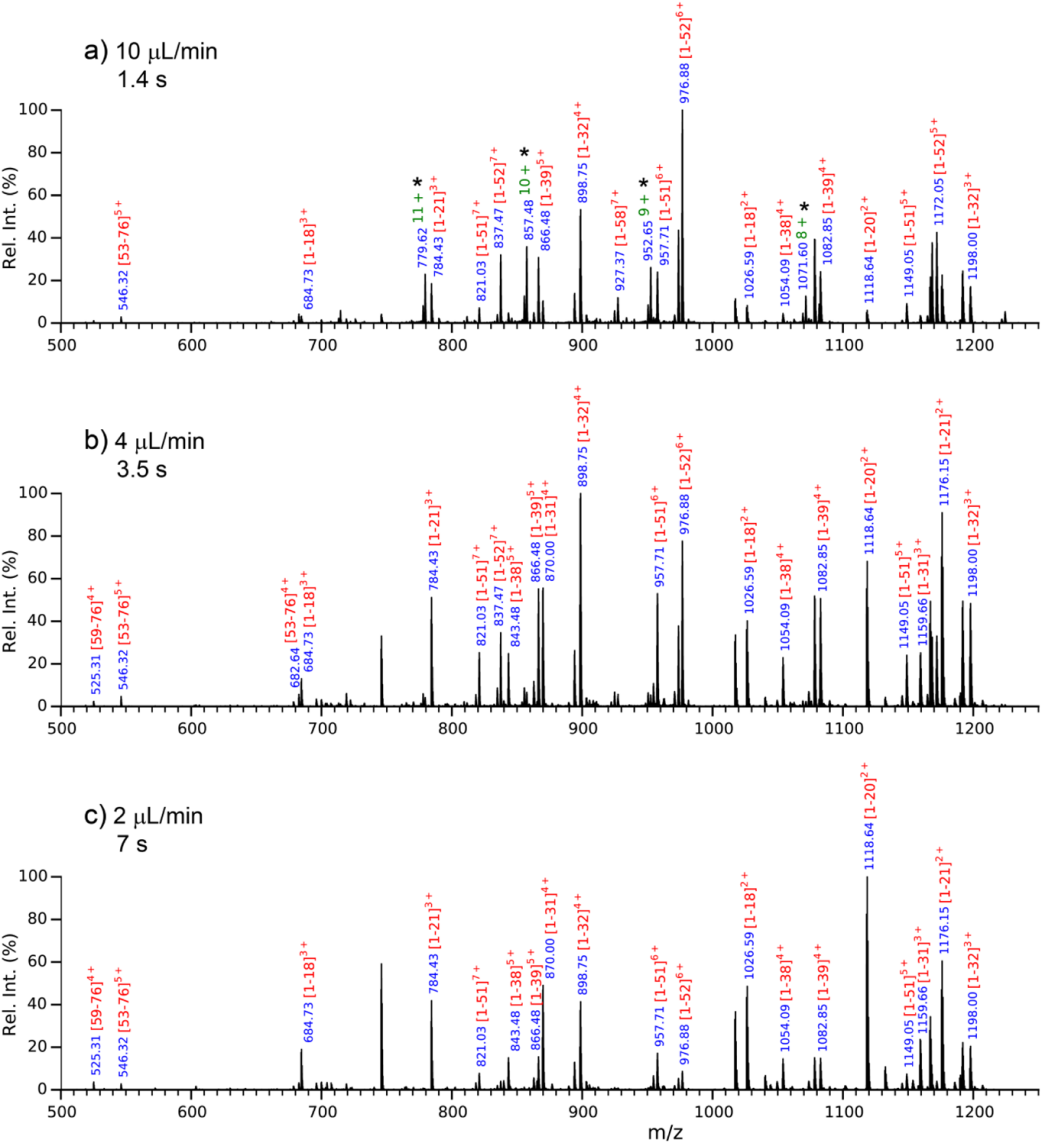
Fig. 9. High-pressure ESI mass spectra of bovine ubiquitin in 2% v/v formic acid under different solution flow rates. a) 10 μL/min, b) 4 μL/min, and c) 2 μL/min. ESI capillary temperate is 220°C for all cases. The corresponding residence time of solution within the heated capillary are 1.4 s (a), 3.5 s (b), and 7 s (c). Asterisk * depicts the peak from intact ubiquitin. Reproduced with permission from J. Mass Spectrom. (2016), 51, 396–411.^73)^

We previously showed that simply heating the ESI capillary would turn it into an on-line digestion reactor. The online operation eliminated the need to transfer the sample from the digestion reactor, and the output of the digestive reaction could be monitored and manipulated on a near-real-time basis.^73,87)^ At optimum temperature, the overall time for digestion and ESI-MS acquisition could be as short as 3 s.^73)^ One example is shown in Fig. 9. The sample solution was rapidly heated when flowing through a heated ESI capillary, and the digestion products were ionized by ESI *in-situ* when the solution emerged from the tip of the heated capillary. The mass spectra of bovine ubiquitin are shown for different solution flow rates and hence different heating times. In all cases, the ESI capillary was kept at 220°C. The peaks originating from the selective digestion products dominate the mass spectrum even with a flow rate of 10 μL/min, which corresponds to a 1.4 s heating time, but there are still traces of intact ubiquitin, labeled with asterisks. The peaks of intact ubiquitin could still be weakly detected at 4 μL/min, but completely disappeared at 2 μL/min. These results showed that simply infusing the peptide/protein solution through a heated capillary at ∼220°C for a few seconds was sufficient to yield digestion products that is enough to provide the coverage for all Asp sites for proteins including ubiquitin and myoglobin. It should be noted that the direct analysis of a large number digested product is relatively difficult and the precise peak annotation is possible only with high resolution MS.

### Online rapid digestion HTLC-HPESI-MS

LC-MS is the standard workflow for the analysis of digestion products in proteomic research. We envision that it is possible to incorporate an online non-enzymatic digestion reactor to the HTLC-HPESI-MS system to further improve the throughput of the proteomics analysis. The digestion reactor can be in the formed of liquid transfer capillary heated by heat block or microwave oven. By placing the heated reactor before the column, the back pressure introduced by the column allows the reactor to be heated to an elevated temperature greater than the column. Since the digestion products were produced at high temperature, they should be compatible with the high-temperature LC as well. The fast separation of HTLC can also keep the cleaved peptides intact owing to short residence time in the hot column. Such an investigation is currently underway in our laboratory.

In conclusion, the trend in the LC development has been toward the use of smaller particles, higher pressure and the use of high temperature to achieve fast separation without sacrificing the plate count. Heat resistive columns and capillary columns have become available commercially to the common user and their stability at high temperature is steadily being improved. Sub-minutes separation under the isocratic mode is now not uncommon, and ultra-fast gradient elution of peptides in less than <30 s has also been achieved by HTLC,^7)^ paving the way towards the ambient LC or ambient LC-MS that produces a near-real-time response like the ambient mass spectrometry. The use of capillary LC is attractive because it requires a low minimum sample amount, achieves high linear velocity at microliter/min volumetric flow rate, and allows the subcritical water LC to be coupled with microflow ESI-MS. High-temperature capillary LC could be coupled directly to MS *via* a super-atmospheric ESI source that prevents the mobile phase from boiling and provide a stable electrospray for a pure aqueous solution without electrical discharge.

## Notes

Mass Spectrom (Tokyo) 2019; 8(2): S0079
